# A systematic review assessing incorporation of prophylactic splenic artery embolisation (pSAE) into trauma guidelines for the management of high-grade splenic injury

**DOI:** 10.1186/s42155-023-00414-6

**Published:** 2023-12-16

**Authors:** Warren Clements, Mark Fitzgerald, S. Murthy Chennapragada, Joseph Mathew, Christopher Groombridge, Ee Jun Ban, Matthew W. Lukies

**Affiliations:** 1 Department of Radiology, Alfred Hospital, 55 Commercial Rd, Melbourne, VIC 3004 Australia; 2 Department of Surgery, Monash University Central Clinical School, Melbourne, Australia; 3 National Trauma Research Institute, Melbourne, Australia; 4 Department of Trauma, Alfred Health, Melbourne, Australia; 5 Discipline of Child and Adolescent Health, Sydney Medical School, Faculty of Health Sciences, University of Sydney, Sydney, NSW Australia; 6 Department of Medical Imaging, The Children’s Hospital at Westmead, Sydney, NSW Australia; 7 Acute General Surgical Unit, Alfred Health, Melbourne, Australia

**Keywords:** Systematic review, splenic, trauma, Guideline, Embolization

## Abstract

**Background:**

Splenic artery embolisation (SAE) has become a vital strategy in the modern landscape of multidisciplinary trauma care, improving splenic salvage rates in patients with high-grade injury. However, due to a lack of prospective data there remains contention amongst stakeholders as to whether SAE should be performed at the time of presentation (prophylactic or pSAE), or whether patients should be observed, and SAE only used only if a patient re-bleeds. This systematic review aimed to assess published practice management guidelines which recommend pSAE, stratified according to their quality.

**Methods:**

The study was registered and reported according to the Preferred Reporting Items for Systematic Reviews and Meta-Analyses (PRISMA) statement. Medline, PubMed, Cochrane, Embase, and Google Scholar were searched by the study authors. Identified guidelines were graded according to the Appraisal of Guidelines Research and Evaluation II (AGREE-II) instrument.

**Results:**

Database and internet searches identified 1006 results. After applying exclusion criteria, 28 guidelines were included. The use of pSAE was recommended in 15 guidelines (54%). This included 6 out of 9 guidelines that were high quality (66.7%), 4 out of 9 guidelines that were moderate quality (44.4%), and 3 out of 10 (30%) guidelines that were low quality, *p* = 0.275.

**Conclusions:**

This systematic review showed that recommendation of pSAE is more common in guidelines which are of high quality. However, there is vast heterogeneity of recommended practice guidelines, likely based on individual trauma systems rather than the available evidence. This reflects biases with interpretation of data and lack of multidisciplinary system inputs, including from interventional radiologists.

**Supplementary Information:**

The online version contains supplementary material available at 10.1186/s42155-023-00414-6.

## Background

The spleen is the most frequently injured abdominal organ after blunt trauma [1]. Splenic injury is commonly graded using the American Association for the Surgery of Trauma (AAST) splenic injury scale, where high-grade injury is defined as AAST IV or V injury [2]. Conservative management of high-grade injury has reported re-bleed rates of as high as 75% in adults [3]. As a part of modernisation of multidisciplinary treatment, splenic artery embolisation (SAE) has become a vital treatment strategy in improving the rate of spleen preservation (aka splenic salvage) in patients with high-grade injury [4], and its use is thus increasing [1].

However, due to a lack of prospective data there remains contention amongst stakeholders as to whether SAE should be performed at the time of presentation (prophylactic or pSAE), or whether patients should be observed and SAE or splenectomy used only if a patient re-bleeds, according to haemodynamics and local resources [5, 6]. The only randomised and controlled trial to date comparing pSAE or a strategy of initial observation, showed no difference in mortality between the groups (0% vs 0.8%). However, the authors showed that pSAE resulted in 100% splenic salvage compared to an observation-first approach of 93.7%, where 32.3% of patients required SAE due to re-bleeding. The authors showed that pSAE resulted in shorter median hospital length of stay (9 vs 13 days) and fewer complications (29.2% vs 41.5%) [6]. Based on this and retrospective studies across the globe [7–12], splenic salvage of AAST IV and V injury in stable patients should be at least 90% at a major trauma centre as a minimum quality benchmark, and pSAE is an effective treatment to achieve this [1, 6, 13].

It is an accepted management practice that when heterogeneity exists in decision-making, agreeing on a treatment guideline has a role in fostering efficient workflow [14]. In many hospitals, guidelines now exist for almost all procedures. Publication of robust guidelines from major and/or notable organisations has immense value as they provide expert-based guidance (ideally based on high-level evidence) on which individual hospitals can mould their daily practice [15]. However, as evidence is available to all, the basis of major decision-making in guidelines should theoretically be similar [16, 17]. However, many institutions continue to propose management approaches based on their own preferences, team structure, and/or interpretation of the available data [18–42].

This study aimed to systematically review existing published splenic trauma management guidelines and assess whether they recommend pSAE in stable patients with high-grade injury, based on the overall guideline quality.

## Methods

### Registration

The study was registered on the PROSPERO database (record number CRD42023440729) and is reported according to the Preferred Reporting Items for Systematic Reviews and Meta-Analyses (PRISMA) statement.

### Data identification and collection

Medline, PubMed, Cochrane, Embase, and Google Scholar were searched by the study authors based against the PICO format (patient, intervention, comparator, outcome), using the following MeSH terms: “Embolization, Therapeutic/methods”[MeSH], “Abdominal Injuries/therapy”[MeSH], “wounds and injuries [MeSH]”, “Embolization, Therapeutic”[MeSH], “splenic artery” [MeSH], “spleen” [MeSH]. The following additional keywords were searched: trauma, embolization, embolisation, angioembolization, angioembolisation, guideline, protocol, pathway, nonoperative, NOM.

### Inclusion and exclusion criteria

Enrolled publications included practice management guidelines from any organisation, for example societies, colleges, government bodies, and hospitals. This included publications where splenic treatment guidelines were presented even if the intention of the study was not specifically to discuss the guideline. All studies within the last 20 years were included (1 January 2003 to 1 January 2023). Duplicate studies were excluded. Studies in the paediatric population were excluded given evidence on trauma management in this context is not comparable to adult populations [43]. Studies were also excluded when they were not in English (*n* = 3), or when the full text could not be obtained (*n* = 1).

### Guideline quality

The Appraisal of Guidelines for Research and Evaluation II (AGREE-II) instrument has been formulated specifically to assist with development and appraisal of guidelines to ensure that clinicians can measure a guideline’s quality before implementing it in daily use [15]. Guidelines were independently evaluated by 2 study investigators (WC and ML) using the 2017 update of the AGREE-II instrument [44] and included grading against 6 different domains. Studies were then graded for quality as high, moderate, or low. Studies were graded as high when they scored greater than or equal to 60% of the maximum score in 3 or more domains, including domain 3 (rigour of development). Studies were graded moderate when they scored greater than or equal to 60% of the maximum score in 3 or more domains, but not including domain 3. Studies were scored as low when they scored less than 60% in 2 or more domains and less than 50% in domain 3. This determination was according to precedent from previous similar studies which have used this tool [43, 45].

### Outcomes

The primary outcome was to assess whether the study recommended the use of pSAE after high grade splenic trauma in stable patients, defined as embolisation (either proximal or distal) for splenic injury of AAST grade IV or V, regardless of the presence of a vascular lesion (active bleeding, arteriovenous fistula, or pseudoaneurysm), in stable patients. Secondary outcomes included stratification of the guideline quality and recommendations according to the type of institution authoring the guideline as well as the region of origin.

### Statistical analysis

Numerical data were presented as percentage when calculated using the AGREE-II instrument, or number (percentage). Where relevant, assessment for differences of proportion between high, moderate, and low-quality guidelines was performed using the Chi Square test in Stata (Version 17.0-BE, StataCorp, Texas, USA). Probability values of less than 0.05 were deemed statistically significant.

## Results

**Fig. 1:**
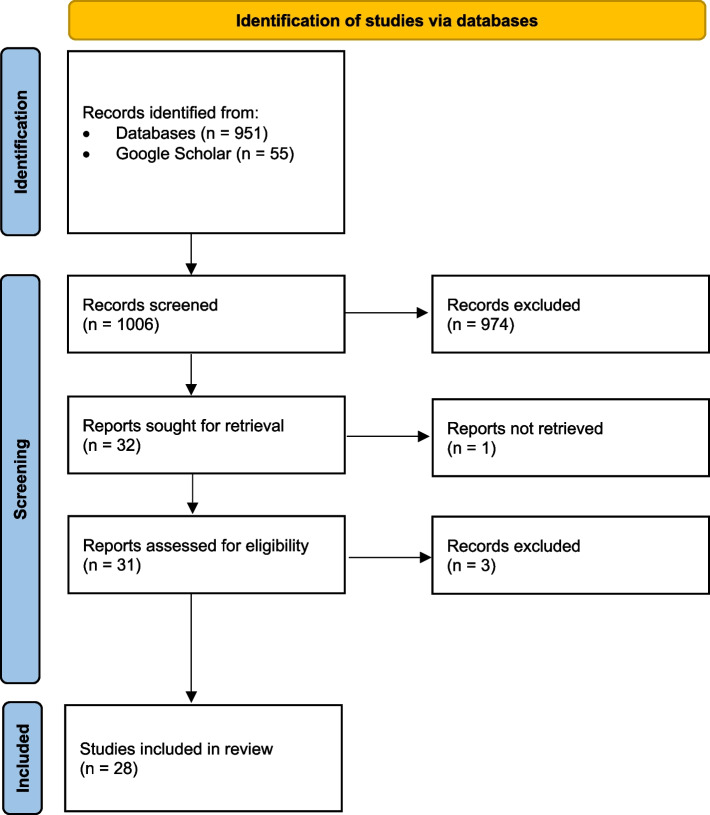
Flow diagram of study selection

Search results were identified from the initial query including 951 from databases plus an additional 55 from Google Scholar. After applying exclusion criteria, a total of 28 published guidelines were included in the analysis as shown in Fig. 1. This included 5 guidelines from medical societies, 1 from a government institution, and 22 from individual hospital networks. The majority of the guidelines were from Europe and Asia (9 each, 32%), followed by North America (8, 29%) and Australia (2, 7%).

Table 1 shows the assessment of guideline quality, where 9 studies were of high quality, 9 studies of moderate quality, and 10 of low quality. As shown in Table 2, 27 out of 28 studies recommended the use of SAE in the management of splenic trauma, the only exception being the study from Koca et al. which did not mention embolisation in their flow chart or text, however, did allude to the concept elsewhere in the document without it being specifically mentioned [36]. In terms of the primary study endpoint, 15 guidelines (54%) recommended pSAE for high-grade injury. This included 6 out of 9 guidelines that were high quality (66.7%), 4 out of 9 guidelines that were moderate quality (44.4%), and 3 out of 10 (30%) guidelines that were low quality, *p* = 0.275.

**Table 1:** Assessment of guideline quality according to the AGREE-II instrument. All scores shown as a percentage of the maximum score

Author	Scope and Purpose	Stakeholder involvement	Rigour of development	Clarity of presentation	Applicability	Editorial independence	Overall assessment^a^
Coccolini et al. [18]	100	53	72	100	66	100	High
Bhullar et al. [19]	86	61	68	75	62	100	High
Stassen et al. [16]	100	69	79	92	86	100	High
Rowell et al. [17]	94	72	75	97	84	100	High
Watson et al. [20]	86	50	64	86	44	95	High
Hameed et al. [21]	100	78	77	94	98	68	High
Cheatham et al. [22]	89	28	73	92	64	36	High
Lin et al. [23]	89	44	65	86	54	95	High
Van Der Cruyssen et al. [24]	83	25	75	83	76	68	High
Tugnoli et al. [25]	75	50	57	67	58	86	Moderate
Gilmore et al. [26]	94	64	56	97	58	50	Moderate
Clements et al. [1]	81	42	58	69	86	100	Moderate
Wu et al. [27]	83	39	55	92	68	68	Moderate
Wu et al. [28]	81	28	58	92	62	95	Moderate
Girard et al. [29]	86	28	50	83	66	100	Moderate
Kanlerd et al. [30]	92	47	56	92	78	100	Moderate
Lee et al. [31]	83	25	53	89	48	91	Moderate
Romeo et al. [32]	67	11	51	58	60	91	Moderate
Mitsusada et al. [33]	58	22	52	69	50	73	Low
Frandon et al. [34]	94	31	42	64	60	100	Low
Singh et al. [35]	61	22	43	78	76	59	Low
Koca et al. [36]	50	14	46	56	58	64	Low
Cameron et al. [37]	19	8	25	81	16	32	Low
Ruscelli et al. [38]	39	22	41	36	40	73	Low
El-Matbouly et al. [39]	47	28	42	83	44	100	Low
Brillantino et al. [40]	89	53	47	56	52	100	Low
Chakraverty et al. [41]	58	28	41	36	60	68	Low
University of Colorado [42]	42	6	22	72	30	36	Low

^a^Overall quality assessment determined according to the following

High: > = 60% in > = 3 domains including domain 3 (rigour of development)

Moderate: > = 60% in 3 domains not including domain 3

Low: < 60% in > = 2 domains and domain 3 < 50%

**Table 2:** Summary of the guideline contents including inclusion of prophylactic splenic artery embolisation

Author	Quality assessment^a^	Year	Digital Object Identifier or Link	Location	Institution of lead author^b^	Recommends prophylactic splenic artery embolisation^c^
Coccolini et al. [18]	High	2017	https://doi.org/10.1186%2Fs13017-017-0151-4	United Kingdom	Society	Yes
Bhullar et al. [19]	High	2017	https://doi.org/10.1097/ta.0000000000001366	United States	Hospital	Yes
Stassen et al. [16]	High	2012	https://doi.org/10.1097/ta.0b013e3182702afc	United States	Society	Yes
Rowell et al. [17]	High	2017	https://doi.org/10.1097/ta.0000000000001323	United States	Society	Yes
Watson et al. [20]	High	2015	https://doi.org/10.1007/s00068-015-0520-1	United States	Hospital	Yes
Hameed et al. [21]	High	2019	http://www.phsa.ca/Documents/Trauma-Services/Spleen%2008%20Full%20CPG%20for%20download.pdf	Canada	Society	No, only if vascular lesion
Cheatham et al. [22]	High	2015	https://surgicalcriticalcare.net/Guidelines/Blunt%20splenic%20injury%202,015.pdf	United States	Hospital	Yes
Lin et al. [23]	High	2022	https://doi.org/10.1007/s00464-022-09531-0	Taiwan	Hospital	No, only if vascular lesion
Van Der Cruyssen et al. [24]	High	2016	https://doi.org/10.1186/s13017-016-0100-7	Belgium	Hospital	No, only if vascular lesion
Tugnoli et al. [25]	Moderate	2015	https://doi.org/10.1007/s00595-014-1084-0	Italy	Hospital	Yes
Gilmore et al. [26]	Moderate	2022	https://www.canberrahealthservices.act.gov.au/__data/assets/word_doc/0006/2074623/Splenic-Trauma.docx	Australia	Government	Yes
Clements et al. [1]	Moderate	2020	https://doi.org/10.1186/s42155-020-00185-4	Australia	Hospital	Yes
Wu et al. [27]	Moderate	2007	http://dx.doi.org/10.1177/000313480707300915	Taiwan	Hospital	No, only if vascular lesion
Wu et al. [28]	Moderate	2008	http://dx.doi.org/10.1007/s00268-007-9322-x	Taiwan	Hospital	No, only if vascular lesion and isolated splenic injury
Girard et al. [29]	Moderate	2016	http://dx.doi.org/10.1016/j.jviscsurg.2016.04.005	France	Hospital	No, only if vascular lesion
Kanlerd et al. [30]	Moderate	2022	https://doi.org/10.1016/j.cjtee.2021.09.006	Thailand	Hospital	Yes
Lee et al. [31]	Moderate	2018	https://doi.org/10.1177/1024907918773202	South Korea	Hospital	No, only if vascular lesion
Romeo et al. [32]	Moderate	2020	https://doi.org/10.1007/s00595-020-02177-2	Italy	Hospital	No, only if vascular lesion
Mitsusada et al. [33]	Low	2014	https://doi.org/10.1002/ams2.37	Japan	Hospital	No, only if vascular lesion
Frandon et al. [34]	Low	2016	https://doi.org/10.1016/j.jviscsurg.2016.04.010	France	Hospital	Yes
Singh et al. [35]	Low	2017	http://dx.doi.org/10.4329/wjr.v9.i4.155	India	Hospital	Yes
Koca et al. [36]	Low	2013	https://doi.org/10.5505/tjtes.2013.89411	Turkey	Hospital	No
Cameron et al. [37]	Low	2013	https://books.google.com.au/books?id = FM6fDQAAQBAJ	United States	Hospital	Yes
Ruscelli et al. [38]	Low	2017	https://www.annaliitalianidichirurgia.it/wp-content/uploads/2018/10/06_2648blocco.pdf	Italy	Hospital	No, only if vascular lesion
El-Matbouly et al. [39]	Low	2016	https://doi.org/10.1016/j.surge.2015.08.001	Qatar	Hospital	No, only if vascular lesion
Brillantino et al. [40]	Low	2016	https://doi.org/10.1007/s00068-015-0575-z	Italy	Hospital	No, only if vascular lesion
Chakraverty et al. [41]	Low	2012	https://doi.org/10.1007/s00270-012-0339-7	United Kingdom	Society	No, only if vascular lesion
University of Colorado [42]	Low	2018	https://medschool.cuanschutz.edu/docs/librariesprovider74/trauma-and-acute-care-surgery-pdfs/trauma-protocols/spleen-trauma-2018.pdf?sfvrsn = e32141b9_2	United States	Hospital	No, only if vascular lesion

^a^According to the classification in Table 1

^b^Defined as the affiliation of the authors, either hospital, society, college, or government

^c^Defined as whether the guideline recommends prophylactic embolisation of high grade splenic trauma (AAST IV or V) regardless of the presence of a vascular lesion

Table 3 shows the demographics of high-quality guidelines and compares them to moderate and low-quality guidelines. High quality guidelines were more commonly published by societies (44.4% vs 0% vs 10%) while both moderate and low-quality guidelines were more likely to be published by hospitals (55.6% vs 88.9% vs 90.0%), *p* = 0.074. In addition, high-quality guidelines were more likely to arise from the North American continent (66.7% vs 0% vs 20%) while both moderate and low-quality guidelines were more likely to arise from Europe (22.2% vs 33.3% vs 40%) or Asia (11.1% vs 44.4% vs 40%), *p* = 0.030. High, moderate, and low-quality guidelines were of similar likelihood to be published in the last 5 years (55.6% vs 55.6% vs 30%, *p* = 0.430).

**Table 3:** Demographics of splenic artery embolisation guidelines according to quality

	High quality guidelines^a^	Moderate quality guidelines^a^	Low quality guidelines	*p*-value
**Total number**	9	9	10	N/A
**Published within the last 5 years (number, percentage)**	5 (55.6%)	5 (55.6%)	3 (30.0%)	0.430
**Institution of lead author**^b^** (number, percentage)**	Society: 4 (44.4%) Hospital: 5 (55.6%) Government: 0 (0%)	Society: 0 (0%) Hospital: 8 (88.9%) Government: 1 (11%)	Society: 1 (10.0%) Hospital: 9 (90.0%) Government: 0 (0%)	0.074
**Continent of origin (number, percentage)**	Europe: 2 (22.2%) Asia: 1 (11.1%) North America: 6 (66.7%) Australia: 0 (0%)	Europe: 3 (33.3%) Asia: 4 (44.4%) North America: 0 (0%) Australia: 2 (22.2%)	Europe: 4 (40%) Asia: 4 (40%) North America: 2 (20%) Australia:0 (0%)	0.030
**Recommends prophylactic splenic artery embolisation**^c^** (number, percentage)**	6 (66.7%)	4 (44.4%)	3 (30.0%)	0.275

^a^According to the classification in Table 1

^b^Defined as the affiliation of the authors, either hospital, society, college, or government

^c^Defined as whether the guideline recommends routine embolisation of high grade splenic trauma (AAST IV or V) regardless of the presence of a vascular lesion

## Discussion

This systematic review identified 28 guidelines on the treatment of blunt splenic injury in adults and of these, only 9 were of high quality according to the AGREE-II instrument. The incorporation of pSAE was seen in 54% which is modest, despite the available evidence [6–12].

In terms of the primary outcome, high-quality guidelines had a higher incorporation of pSAE (66.7%) and while the difference between high, moderate, and low-quality was not statistically significant, it is likely due to a type 2 error from a small sample. High-quality guidelines were also more likely to be written by societies while moderate and low-quality guidelines were more likely written by individual hospitals. Societies may be more likely to consider the value of their brand endorsement, and thus likely to consider the importance of stakeholder engagement and input during development. High-quality guidelines were also likely to arise from North America, however this may be confounded as this was also the location of many leading trauma societies, and the origin of the AGREE-II Enterprise. This may reflect the matured systems of trauma within a continent where trauma has a high prevalence. The authors strongly recommend that anyone who develops or updates a clinical practice guideline considers the AGREE-II instrument, or other similarly validated tools, to ensure that the standard of their recommendations are transparent and robust. From the results of this study, it is felt unlikely that any of the guidelines have specifically used such tools.

Only 5 societies worldwide have chosen to publish a guideline, and some are well overdue for modernisation. An example is the guideline from the Cardiovascular and Interventional Society of Europe (CIRSE), the largest IR society in the world. The current guideline is now over 10 years old and devoid of robust evidence, detail, clarity, and applicability [41], not in keeping with the quality usually seen from such an influential organisation. The low overall uptake of pSAE will be improved if IRs and IR societies such as CIRSE and the Society of Interventional Radiology (SIR), take a larger role in the governance of trauma, integrating themselves in trauma networks, and aligning themselves with local IR and trauma societies in different regions. It is also recommended that societies with sufficient infrastructure consider developing and regularly updating guidelines to remain relevant with constantly changing literature.

In general, most guidelines performed poorest in describing their stakeholder engagement (even those which were high quality), rarely involving a patient advocate, and often missing input from a wider multidisciplinary team. This opens the guideline to bias with reader interpretation and thus implementation of the recommendations. While this may have been acceptable in the early days of trauma which was typically run by surgical specialties, modern trauma management involves centralised, co-ordinated, tertiary care and involves a range of key stakeholders including emergency, surgery, interventional radiology, diagnostic radiology, anaesthetics, intensive care, and many others. In general, guidelines also performed generally poorly on applicability, often presenting an ideal pathway, but omitting measures to ensure that it is feasible, ways to overcome challenges to feasibility, costs, and auditing. It should be acknowledged that some studies presenting a guideline as a smaller component of a wider clinical investigation may not have felt the need to describe their guideline development in detail, and they were still included in this analysis. However, the robust development of a guideline has direct relevance on the downstream utility and it should be encouraged that authors present this vital data moving forward.

The domain of clarity of presentation had a somewhat dichotomous result. Those that published a clear and relevant flow chart and/or used an executive summary at the beginning generally scored highly. However, those that described their treatment without a specific chart scored lower. In addition, editorial independence was generally transparent through mandated reporting standards in journals. Those that did not publish their guideline in a journal often did not choose to voluntarily report any potential conflicts of interest.

A major component of the interpretation bias of guidelines is the failure for treatment pathways to adequately consider splenic function and instead place a weighted focus on mortality as the only endpoint [4]. This is also in part because it is difficult to measure treatment success in trauma and most studies in this context are retrospective with several inherent biases, leading to scepticism of results and lack of applicability of approaches where trauma systems aren’t replicable [6, 8]. In addition to these challenges, interventional radiology is still a young specialty, and as such training and governance structures vary significantly between different hospitals, regions, and countries [5]. This means that access to expert skills with appropriate training is also not universal.

Given SAE was mentioned in all guidelines, trauma networks should endeavour to include a sustainable IR service to be involved in the management of major trauma. This should involve expansion of the current IR workforce so that smaller hospitals have timely IR services available, or for patients to be sent to a centre with resources to prioritise splenic salvage *in addition* to mortality prevention. The benefits of salvage are vast and include the avoidance of a laparotomy with associated morbidity, prevention of overwhelming post-splenectomy infection (OPSI), reduced need for future vaccinations, reduced need for future prophylactic antibiotics, and cost savings to the individual and society [46–49].

The authors acknowledge that this study is limited by the interpretation and assessment of guideline quality and while this is an objective validated tool, still requires individual interpretation. In addition, guidelines published within a clinical cohort study may not have provided the full breadth of information to allow for their interpretation as discussed earlier. However, the authors chose to present all guidelines rather than limit the analysis to purely those from major societies given the very small sample. The statistical analysis in Table 3 is also based on a small sample. The guidelines also cross the 2018 update to the AAST injury grading criteria [2] and this may theoretically affect management decisions moving forward for those with an earlier guideline based on the 1994 AAST definitions. There is also variation in how stakeholders may view and define “prophylactic embolisation” with differing opinions on the relative importance of parenchymal injury and vascular lesions.

## Conclusions

This systematic review showed that recommendation of pSAE is more common in guidelines which are of high quality. However, there is vast heterogeneity of recommended practice guidelines, likely based on individual trauma systems. This reflects biases with interpretation of data, and lack of multidisciplinary system inputs from IRs. More societies should publish guidelines, ensuring they are high-quality by conforming to existing validated reporting standards. Centres or countries which do not have the infrastructure to support pSAE are encouraged to embed IR within their trauma governance structure and overcome barriers to implementation to improve quality of care, rather than using a self-generated treatment algorithm which may not provide their patients with the established short- and long-term benefits of splenic salvage.

### Supplementary Information

**Additional file 1: Supplement 1.** Database search terms and results.

## Data Availability

The datasets generated and/or analysed during the current study are not publicly available but are available from the corresponding author on reasonable request. Please contact the corresponding author for additional information.

## Abbreviations

IR: Interventional radiology
AAST: American association for the surgery of trauma
SAE: Splenic artery embolisation
pSAE: Prophylactic splenic artery embolisation
OR: Odds ratio
AGREE-II: Appraisal of guidelines for research and evaluation II
PRISMA: Preferred reporting items for systematic reviews and meta-analyses
PICO: Patient, intervention, comparator, outcome
CIRSE: Cardiovascular and interventional society of europe
SIR: Society of interventional radiology
OPSI: Overwhelming post-splenectomy infection
