# Supplementary figures and images for: A systematic review assessing incorporation of prophylactic splenic artery embolisation (pSAE) into trauma guidelines for the management of high-grade splenic injury
Source: CVIR Endovasc. 2023 Dec 16;6:62. doi: 10.1186/s42155-023-00414-6 (PMC10725392; doi:10.1186/s42155-023-00414-6)

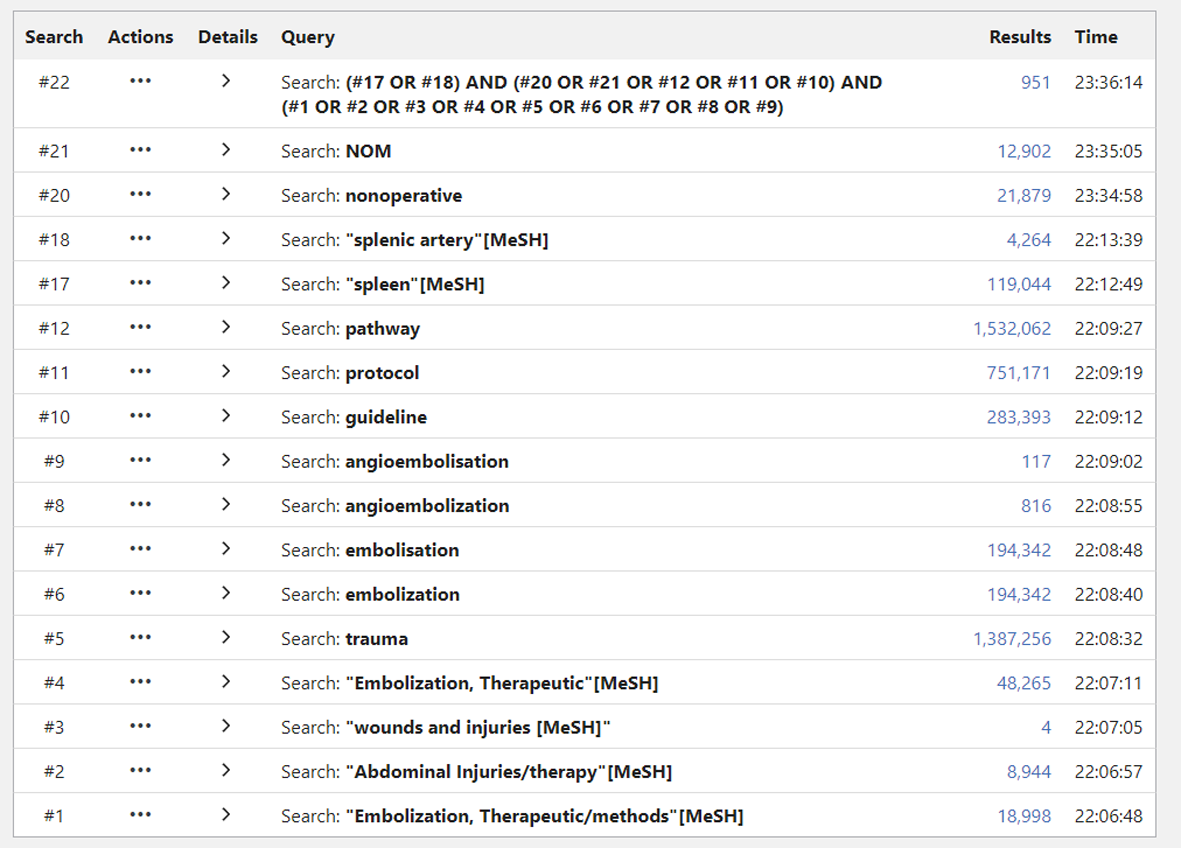

Supplement: Supplementary file 1 — Additional file 1: Supplement 1. Database search terms and results. [file 42155_2023_414_MOESM1_ESM.tif]
